# Exploring the Microbial Community and Functional Characteristics of the Livestock Feces Using the Whole Metagenome Shotgun Sequencing

**DOI:** 10.4014/jmb.2209.09013

**Published:** 2022-12-16

**Authors:** Hyeri Kim, Eun Sol Kim, Jin Ho Cho, Minho Song, Jae Hyoung Cho, Sheena Kim, Gi Beom Keum, Jinok Kwak, Hyunok Doo, Sriniwas Pandey, Seung-Hwan Park, Ju Huck Lee, Hyunjung Jung, Tai Young Hur, Jae-Kyung Kim, Kwang Kyo Oh, Hyeun Bum Kim, Ju-Hoon Lee

**Affiliations:** 1 Department of Animal Resources Science, Dankook University, Cheonan, Republic of Korea; 2 Department of Animal Science, Chungbuk National University, Cheongju, Republic of Korea; 3 Division of Animal and Dairy Science, Chungnam National University, Daejeon, Republic of Korea; 4 Korean Collection for Type Cultures, Korea Research Institute of Bioscience and Biotechnology (KRIBB), Jeongeup, Republic of Korea; 5 Animal Nutrition & Physiology Division, National Institute of Animal Science, RDA, Wanju, Republic of Korea; 6 Animal Diseases & Health Division, National Institute of Animal Science, RDA, Wanju, Republic of Korea; 7 Advanced Radiation Technology Institute, Korea Atomic Energy Research Institute, Jeongeup, Republic of Korea; 8 Microbial Safety Division, National Institute of Agricultural Sciences, Rural Development Administration, Wanju, 55365, Republic of Korea; 9 Department of Food Animal Biotechnology, Department of Agricultural Biotechnology, Center for Food and Bioconvergence, Seoul National University, Seoul 08826, Republic of Korea

**Keywords:** Foodborne pathogens, livestock, livestock environment, metagenome shotgun sequencing, potential virulence

## Abstract

The foodborne illness is the important public health concerns, and the livestock feces are known to be one of the major reservoirs of foodborne pathogens. Also, it was reported that 45.5% of foodborne illness outbreaks have been associated with the animal products contaminated with the livestock feces. In addition, it has been known that the persistence of a pathogens depends on many potential virulent factors including the various virulent genes. Therefore, the first step to understanding the public health risk of livestock feces is to identify and describe microbial communities and potential virulent genes that contribute to bacterial pathogenicity. We used the whole metagenome shotgun sequencing to evaluate the prevalence of foodborne pathogens and to characterize the virulence associated genes in pig and chicken feces. Our data showed that the relative abundance of potential foodborne pathogens, such as *Bacillus cereus* was higher in chickens than pigs at the species level while the relative abundance of foodborne pathogens including *Campylobacter coli* was only detected in pigs. Also, the microbial functional characteristics of livestock feces revealed that the gene families related to “Biofilm formation and quorum sensing” were highly enriched in pigs than chicken. Moreover, the variety of gene families associated with “Resistance to antibiotics and toxic compounds” were detected in both animals. These results will help us to prepare the scientific action plans to improve awareness and understanding of the public health risks of livestock feces.

## Introduction

The foodborne illnesses constitute public health concerns worldwide and the livestock feces are known to be one of the major reservoirs of foodborne pathogens. It has been shown that 45.5% of foodborne disease outbreaks have been associated with the animal products contaminated with the livestock feces. In addition, it has been known that the persistence of a pathogens depends on a variety of potential virulent factors including the various virulent genes [1].

The persistence of a pathogens depends on many potential virulent factors including the various virulent genes. The genes for potential virulence factors are expressed to colonize, evade, or inhibit the immune responses into the host, enter into or exit the host cell, and acquire a nutrition from the host. Furthermore, pathogenicity of pathogens were also affected by the several factors such as adhesion, invasion, iron acquisition, flagellum, secretion, and toxin production [2]. For example, *Staphylococcus aureus* adheres and invades host epithelial cells using a variety of molecules, such as microbial surface components recognizing adhesive matrix molecules (MSCRAMM) which is important for adhesion and attachment to host cells [3, 4]. In addition, it has been known that the microbial adaptation, such as biofilm formation and movement of DNA mediated by plasmid, mobile DNA elements and transposons were also attributed to resistance to antibiotics [5]. Therefore, the first step to understanding the public health risk of livestock feces is to identify and describe microbial communities and potential virulent genes that contribute to bacterial pathogenicity. The purpose of this study was to evaluate the prevalence of foodborne pathogens and to characterize the virulence associated genes in pig and chicken feces.

## Materials and Methods

### Fecal Sampling

Fecal samples were randomly collected from the rectum of 3 pigs and 3 chickens from the farms, in Chungcheong province South Korea (Table S1). All fecal samples were collected directly from the rectum of pigs and cloaca of chickens, then the fecal samples were placed in sterile test tubes and stored at -80°C to maintain stability of microbiota in the feces. We used pig and chicken fecal samples collected from the farms in Chungcheong province South Korea because the pig and chicken are the two most common livestock types in South Korea. Even though, their geographic distribution varies, Chungcheong province is the province where these animals are raised in the highest density in South Korea.

### DNA Extraction

Total DNA representing the bacterial community was extracted from 200 mg of feces samples using QIAamp FAST DNA Stool Mini kit (Qiagen, Germany), according to the manufacturer’s instructions with a modification. Briefly, cell lysis was conducted with bead-beating the samples twice for 2 min at 300 ×*g* instead of vortex during the DNA extraction process. The samples were incubated for 5min in a water bath at 70°C between beatings. The isolated DNA quantification was conducted using a Colibri Microvolume Spectrometer (Titertek Berthold, Germany) with OD260/280 ratio. Only DNA samples with OD260/280 ratio between 1.80 and 2.00 were used for the whole metagenome shotgun sequencing.

### Whole metagenome Shotgun Sequencing

For the whole metagenome shotgun sequencing, DNA representing the fecal microbial communities extracted from the feces was sequenced using paired-end shotgun sequencing using the Illumina NovaSeq platform at Macrogen Inc. (Korea).

### Whole Metagenome Shotgun Sequence Analysis

Whole metagenome shotgun sequencing was performed to evaluate the prevalence of foodborne pathogens and to characterize the virulence associated genes in pig and chicken feces. To analyze whole metagenomic sequence data from pigs and chickens, the raw sequence data in FASTQ format were submitted to the Metagenomics Rapid Annotation using Subsystem Technology (MG-RAST) pipeline for microbial functional analysis. The quality control of the raw data included the removal of artificial sequences as previously described,[6] and removal of low quality sequences using a modified Dynamic trim[7] for sequences with 5 bp below a 15 Phred score [8]. All the sequence reads were assembled and normalized in MG-RAST. The MG-RAST pipeline uses DESeq to analyze sequence count data, and to remove aspects of inter sample variability caused by differences in sequencing depth of samples. The MG-RAST pipeline uses bowtie to remove sequence reads that match with the genome of the host. The functional annotation and taxonomy identification of the sequence reads was performed using the SEED Subsystems database and Greengenes, a collection of functionally related protein families. Similarity search between sequence reads and the SEED databases was conducted by using an E-value of less than 1 × 10−5, minimum identity of 60%, and a minimum alignment length of 15 amino acids for protein. Multiple t-tests were used to identify significant differences in functional profiles between pigs and chickens using STAMP and GraphPad Prism version 7.00 (USA).

## Results

### The Taxonomic Classification of the Sequences

**Fig. 1:**
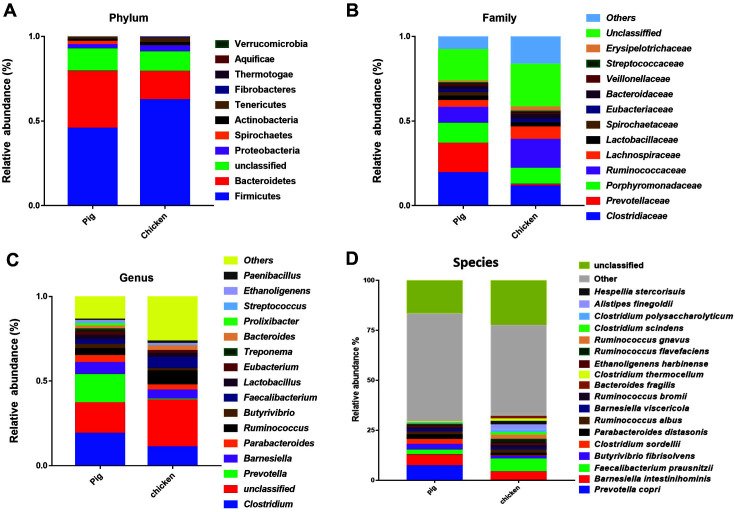
Relative abundance of sequences at the (A) phylum, (B) family, (C) genus, and (D) species levels. Phylogenetic assignment and classification based on 16S rRNA sequence similarity were conducted by using the Greengenes classifier implemented in MG-RAST.

The raw data from Illumina Next sequencing generated an average number of bases of 8,244,358,450, and average number of reads of 54,598,400 per sample (Table S2). After quality trimming, an average of 547,902 contigs were assembled from six feces samples (Table S3). After taxonomy identification using MG-RAST, the result showed that Firmicutes was the most abundant phylum with the relative abundances of 46% and 62% in the fecal samples of pig and chicken, followed by Bacteroidetes in pig (34%) and chicken (17%). Additionally, at the family level, *Clostridiaceae* (19%) was highly present in pig samples, and *Prevotellaceae* (17%), *Porphyromonadaceae* (12%), and *Ruminococcaceae* (9%) showed the lower relative abundances. For the chicken samples, taxonomic classification showed that *Ruminococcaceae* (17%) had the highest relative abundance, followed by *Clostridiaceae* (12%), *Porphyromonadaceae* (9%) and Lachnospiraceae (7%). At the genus levels, *Clostridium* genera were highly abundant in the pig (19%) and chicken (11%) samples. However, the second highly abundant genus was different, *Prevotella* (17%) was present in the pig samples and *Ruminococcus* (8%) in the chicken samples (Fig. 1). At the species level, *Barnesiella intestinihominis* and *Faecalibacterium prausnitzii* were the most abundant species in pig and chicken samples. In pig samples, the relative abundances of *B. intestinihominis* and *Faecalibacterium prausnitzii* were 5.53% and 2.63%, respectively. The relative abundances of *B. intestinihominis* and *Faecalibacterium prausnitzii* were 4.39% and 6.62% in chicken samples. Other than that, *Butyrivibrio fibrisolvens* (2.59%) in pig samples and *Alistipes finegoldii* (2.36%) in chicken samples have been discovered. In addition, a variety of foodborne pathogens were identified at the species level, and the presence of potential foodborne pathogens were also confirmed. Although not large percentage of food poisoning bacteria were found, several food poisoning bacteria were found in pigs and chickens. The relative abundance of *Bacillus cereus* was 0.04% and 0.07% in pig and chicken samples, respectively. The relative abundance of *Clostridium perfringens* was similar in both pig (0.23%) and chicken (0.27%) samples. However, *Campylobacter coli* (0.04%), *Shigella dysenteriae* (0.38%), *Streptococcus pneumonia* (0.23%), *Streptococcus pyogenes* (0.04%) were present in the only pig samples.

### Microbial Functional Characteristics of Livestock Feces Metagenome associated with “Membrane Transport”

**Fig. 2:**
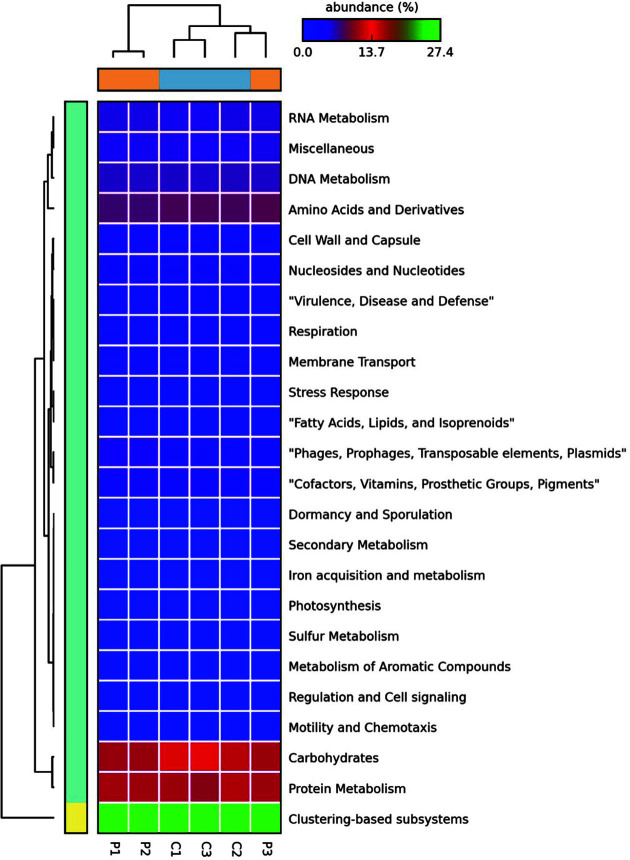
Heatmap of relative abundance of sequences at the level 1 SEED subsystem based on whole metagenome shotgun sequencing data. The e-value cutoff for metagenomics sequence matches to the SEED subsystem database was 1 × 10^-5^ with a minimum alignment length of 15 amino acids. The two-way hierarchical cluster analysis was performed using unweighted pair group method with arithmetic mean (UPGMA) method.

After microbial functional analysis of the sequences, 24 level 1 SEED subsystems were identified in both pig and chicken feces metagenome (Fig. 2).

**Fig. 3:**
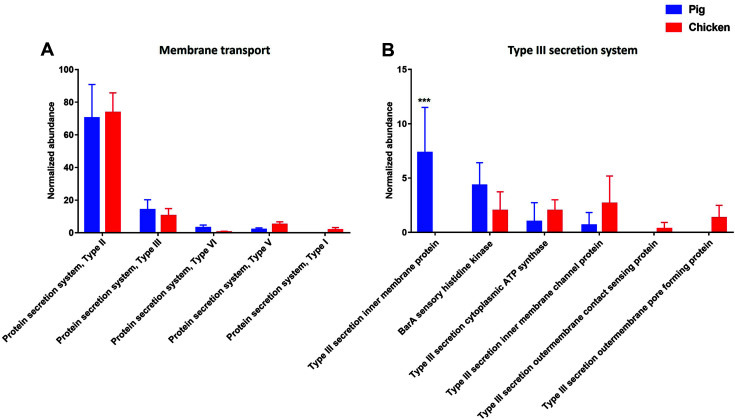
Comparison of the functional capacities of the gut microbiomes between pig and chicken associated with “Membrane transport”. Normalized abundance of the level 2 SEED subsystem classified reads associated within the level 1 SEED subsystem “Membrane transport” (**A**). Normalized abundance of the level 4 SEED subsystem classified reads associated with “Protein secretion system, Type III” (**B**). The error bars show the calculated standard deviation of four replicates, and the [*p* < 0.001], [*p* < 0.01] and [*p* < 0.05] were indicated as [***], [**] and [*], respectively.

The gene families related to “protein secretion system” were identified in the fecal samples at the level 2 SEED subsystems within the level 1 SEED subsystem “membrane transport”. The gene families associated with the Type II, III, V, and VI secretion systems (T2SS, T3SS, T5SS, and T6SS) were present in both pig and chicken samples. However, the gene families related with the Type I secretion system (T1SS) was detected only in chicken samples at the level 2 SEED subsystem (Fig. 3A).

At the level 4 SEED subsystem, the gene families associated with the “Type III secretion inner membrane protein” were detected only in pig samples. However, the gene families related with the “Type III secretion outer membrane contact sensing protein” and “Type III secretion outer membrane pore forming protein” were only detected in the chicken samples. The gene families related with “BarA sensory histidine kinase”, “Type III secretion cytoplasmic ATP synthase”, “Type III secretion inner membrane channel protein” were present in both animals (Fig. 3B).

### Microbial Functional Characteristics of Livestock Feces Metagenome associated with “Phage, Prophage, Transposon Element and Plasmid” and “Quorum Sensing and Biofilm Formation”

Horizontal gene transfer between bacteria has a great impact on the evolution of bacterial pathogens. Phages, plasmids, and pathogenicity islands (PIs) have been recognized as mobile carriers of virulence-associated gene clusters [9].

**Fig. 4:**
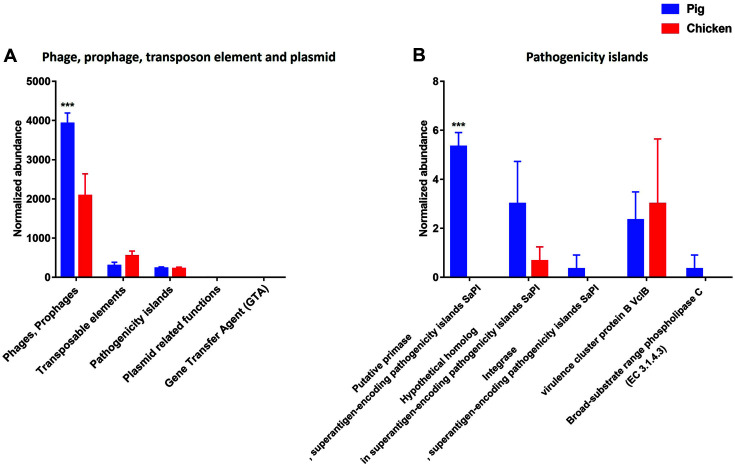
Comparison of the functional capacities of the gut microbiomes between pig and chicken associated with “phage, prophage, transposon element and plasmid”. Normalized abundance of the level 2 SEED subsystem classified reads associated with “Phage, prophage, transposon element and plasmid” (**A**). Normalized abundance of the level 4 SEED subsystem classified reads associated with “Pathogenicity islands” (**B**). The error bars show the calculated standard deviation of four replicates, and the [*p* < 0.001], [*p* < 0.01] and [*p* < 0.05] were indicated as [***], [**] and [*], respectively.

At the level 1 subsystem, the microbial metagenome associated with “Phage, prophage, transposon element and plasmid” covered 3.64% of the total sequences assigned to SEED subsystems. The gene families involved with “Phages, Prophages”, “Transposable elements”, and “Pathogenicity islands” were detected in both pig and chicken samples at the level 2 SEED subsystem. Especially, the functional characteristics of the sequences at the level 2 SEED subsystems showed that the gene families of “Phages, prophages” were significantly enriched in the pig than chicken samples (Fig. 4A). At the level 4 SEED subsystem within the “Pathogenicity islands”, gene families associated with “Putative primase, superantigen-encoding pathogenicity islands SaPI”, “Integrase, superantigen-encoding pathogenicity islands SaPI” and “Broad-substrate range phospholipase C” were only detected in pig samples (Fig. 4B).

**Fig. 5:**
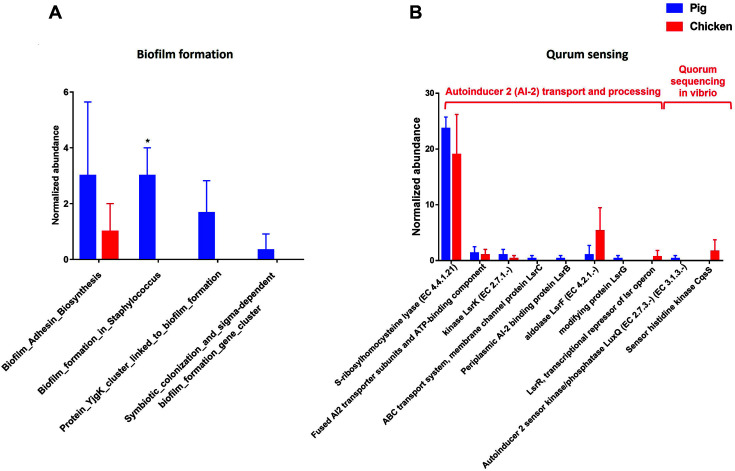
Comparison of the functional capacities of the gut microbiomes between pig and chicken associated with “Quorum sensing and biofilm formation”. Normalized abundance of the level 3 SEED subsystem classified reads associated with “Biofilm formation” (**A**). Normalized abundance of the level 4 SEED subsystem classified reads associated with “Quorum sensing” (**B**). The error bars show the calculated standard deviation of four replicates, and the [*p* < 0.001], [*p* < 0.01] and [*p* < 0.05] were indicated as [***], [**] and [*], respectively.

Microbial functional characteristics of the sequences showed that the gene families related with “Biofilm formation” were highly enriched in pig samples than chicken samples at the level 3 SEED subsystem. At the level 3 SEED subsystem within the level 2 subsystem of “Quorum sensing and biofilm formation”, the gene families involved with “Biofilm Adhesin Biosynthesis”, “Biofilm formation in *Staphylococcus*”, “Protein YjgK cluster linked to biofilm formation” and “Symbiotic colonization and sigma-dependent biofilm formation gene cluster” were highly enriched in pig than chicken samples (Fig. 5A). Furthermore, there were 8 gene families were detected at the level 4 SEED subsystem within the level 3 SEED subsystem of “Autoinducer 2 (AI-2) transport and processing”, and 2 gene families were identified at the level 4 SEED subsystem within the level 3 SEED subsystem “Quorum sequencing in vibrio”. All of the gene families except for “LsrR, transcriptional repressor of Isr operon” were present in pig samples at the level 4 SEED subsystem within the level 3 subsystem “Autoinducer 2 (AI-2) transport and processing”. The gene families associated with “Sensor histidine kinase CqsS” at the level 4 SEED subsystem within the level 3 subsystem “Quorum sequencing in vibrio” were present in only chicken samples (Fig. 5B).

### Microbial Functional Characteristics of Livestock Feces Metagenome associated with “Virulence, Disease and Defense”

**Fig. 6:**
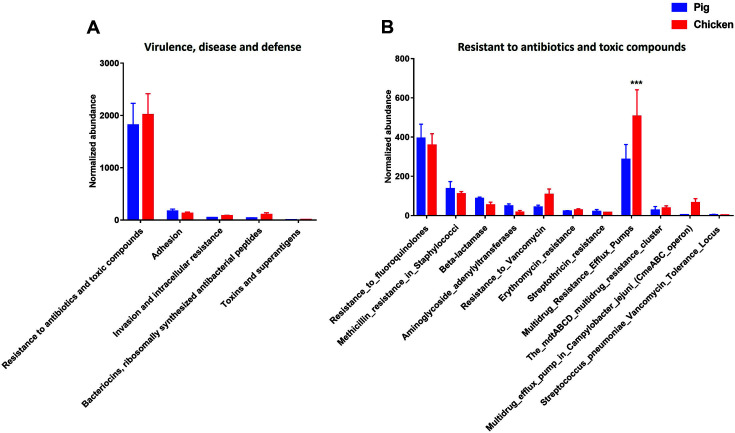
Comparison of the functional capacities of the gut microbiomes between pig and chicken associated with “Virulence, disease and defense”. Normalized abundance of the level 2 SEED subsystem classified reads associated with “Virulence, disease and defense” (**A**). Normalized abundance of proteins at the level 3 SEED subsystem associated with “Resistance to antibiotics and toxic compounds” (**B**). The error bars show the calculated standard deviation of four replicates, and the [*p* < 0.001], [*p* < 0.01] and [*p* < 0.05] were indicated as [***], [**] and [*], respectively.

Microbial functional characteristics of livestock feces metagenome showed that the gene families related with “Resistance to antibiotics and toxic compounds” were the most enriched among the level 2 SEED subsystem categories within the level 1 SEED subsystem named “Virulence, disease and defense” (Fig. 6A). At the level 3 SEED subsystem within the level 2 SEED subsystem “Resistance to antibiotics and toxic compounds”. the gene families related with the resistance to antibiotics, such as Fluoroquinolones, Methicillin, Beta-lactamase, Aminoglycoside, Vancomycin, Erythromycin and Streptothricin were identified in both pig and chicken samples. Especially, chicken samples showed significantly high abundance of gene families “Multidrug Resistance Efflux Pumps” than pig samples at the level 3 SEED subsystem (*p* < 0.01) (Fig. 6B).

**Fig. 7:**
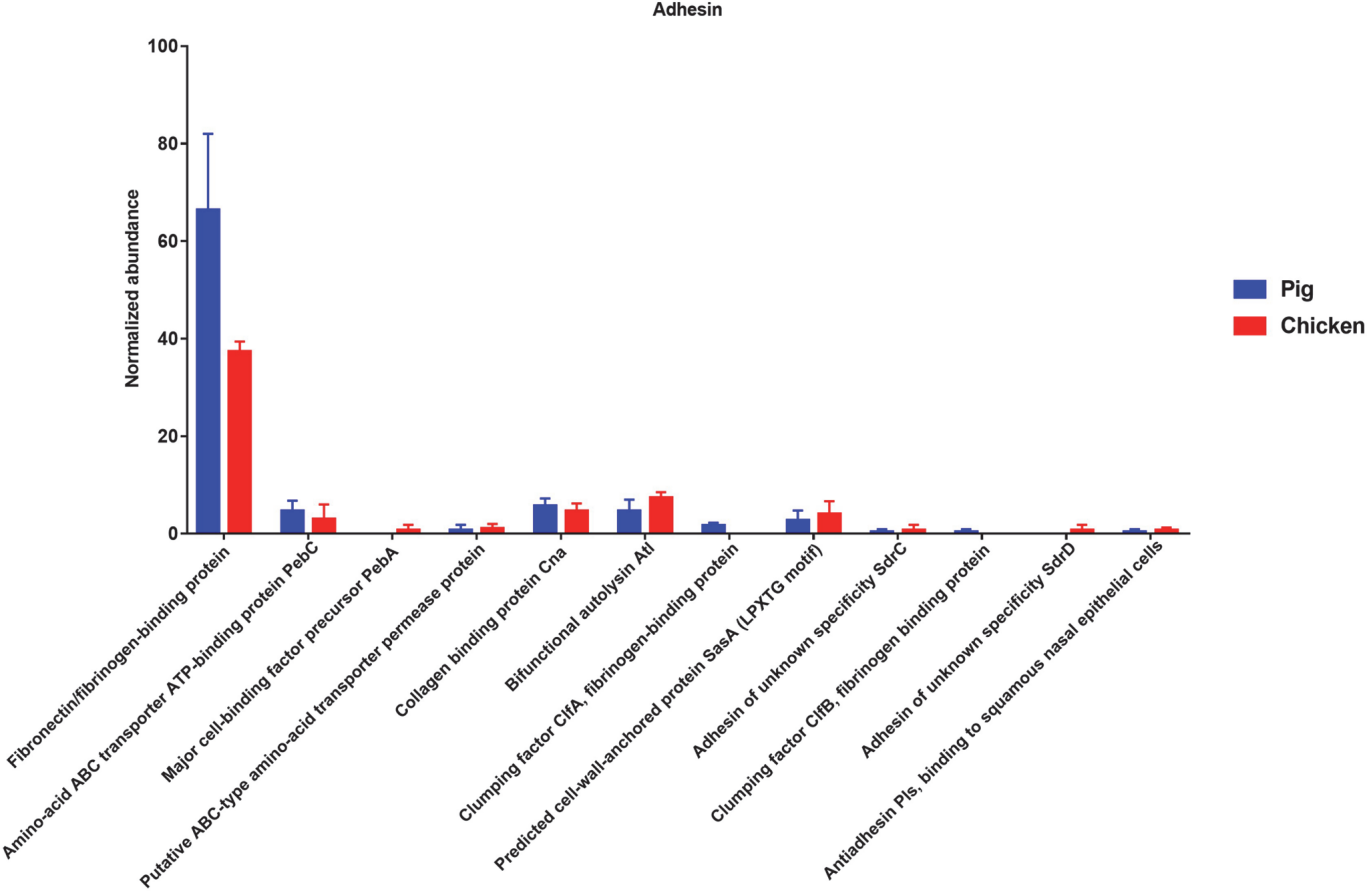
Comparison of the functional capacities of the gut microbiomes between pig and chicken associated with “Virulence, disease and defense”. Normalized abundance of the level 4 SEED subsystem classified reads associated with “Adhesion”. The error bars show the calculated standard deviation of four replicates, and the [*p* < 0.001], [*p* < 0.01] and [*p* < 0.05] were indicated as [***], [**] and [*], respectively.

Regarding the adhesion, a variety of gene families were identified at the level 4 SEED subsystem within the level 3 subsystems “Adhesins of *Campylobacter*” and “Adhesins of *Staphylococcus*”. Especially, the gene families related with “Fibronectin/fibrinogen-binding protein” were highly enriched in pig than chicken samples (*p* < 0.01) (Fig. 7).

## Discussions

Although not large percentage of food poisoning bacteria were found, several food poisoning bacteria were found in both pigs and chickens at the species level. In this study, *C. perfringens* was detected in both pig (0.23%) and chicken samples (0.27%). Furthermore, *C. coli* (0.04%) was present in the pig samples only. *Campylobacter* spp. are members of the family *Campylobacter*iaceae and one of the most common causes of diarrheal foodborne illness [10]. EU data reported that *C. coli* was identified in 13.1%, 9.5% and 87.1% of the isolates from the broilers, cattle, and pigs [11]. As such, our results also showed the presence of *C. coli* in pig samples. These pathogenic bacteria are carried in the animal intestine and shed in the feces. Then this pathogen can be transmitted to humans [12]. Therefore, our results along with others emphasize the good sanitation practices on the farm is an important point to prevent transmission of the pathogens from the perspective of One health. Other than well-known foodborne pathogens, some potential foodborne bacteria were identified in pig and chicken samples of this study. *B. cereus* was present in both pig (0.04%) and chicken (0.07%). However, *S. dysenteriae* (0.38%), *S. pneumonia* (0.23%), and *S. pyogenes* (0.04%) were present in the pig samples only. Zoonotic potential of pathogens in livestock feces is a serious concern, although it is difficult to define these bacteria [13]. Despite the importance of infectious diseases to animal and human, zoonotic potential of infectious diseases of swine and chicken has not been characterized yet. Therefore, there is an urgent need to evaluate the potential zoonotic pathogens in food-producing animals for the prevention of foodborne diseases.

Bacteria have evolved a wide variety of secretion systems that translocate substrates, such as small molecules and proteins. These substrates have functions in the response of a bacterium to its environment and several physiological processes including adhesion, adaptation, survival. and pathogenicity [14]. Thus, it is important to monitoring the membrane transport which possess the secretion systems in the livestock feces samples. Many pathogenic bacteria utilize protein secretion systems to secrete virulence factors from the cytosol of the bacteria into host cells causing mammalian infection [15]. Some of T1SS systems from gram-negative bacteria are often related to virulence such as hemolysin A which cause lysis of red blood cells by disrupting the cell membrane [16]. Furthermore, T1SS is closely associated with the resistance–nodulation–division (RND) family of multidrug efflux pumps. The RND pumps allow bacteria to remove antibacterial compounds out of the cell [17]. In this study, we confirmed the presence of the gene families related with T1SS only in chicken samples at the level 2 SEED subsystem, indicating that the bacteria possessing multidrug efflux pumps could be present in chicken fecal samples. T2SSs found in *Vibrio cholera*, enterotoxigenic *Escherichia coli* (ETEC) and *Pseudomonas aeruginosa* secrete proteins from the periplasm into the extracellular environment [14, 18, 19]. The T6SS which is detected in all of the livestock samples in this study is also a characterized secretion system that appears to constitute a phage-tail-spike-like injectisome, which translocate toxic effector proteins directly into cytoplasm of host cells and induce a pivotal role in pathogenesis and bacterial competition. It is reported that T6SS is required for virulence in human and animal for the pathogens such as *V. cholerae*, *Edwardsiella tarda*, *P. aeruginosa*, *Francisella tularensis*, and *Burkholderia mallei* [20-22]. In this study, we confirmed that the gene families associated with T2SS and T6SS were found in chicken and pig samples at the level 2 SEED subsystem, indicating that the bacteria possessing general secretory pathway (Gsp) and phage-tail-spike-like injectisome could be present in both chicken and pig fecal samples [23]. T3SSs are widespread and allows to inject toxins directly into the cytoplasm of eukaryotic cells. T3SS are found in various foodborne pathogen such as enteropathogenic *E. coli* (EPEC), *Salmonella*, *Pseudomonas*, *Shigella* and *Yersinia* [24]. These specialized protein delivery machines promote the transfer of bacterial effector proteins to the cytoplasm or the plasma membrane of target eukaryotic cells [25, 26]. *Shigella* spp. has Mix-spa gene also called type 3 secretion system (T3SS), and the effector proteins have important functions in invasion and cell-to-cell spreading of bacterial cells in the intestinal epithelium. The proteins forming the *S. dysenteriae* T3SS and all of its effectors are encoded on a large virulence plasmid [27]. In this study, *S. dysenteriae* were detected in only pig samples, which can be linked to the level 4 SEED subsystem, Type 3 secretion inner membrane protein which was found in pig samples only. In this study, it was confirmed that the gene families associated with the bacterial secretion system were present in pig and chicken feces using the whole metagenome shotgun sequencing of livestock fecal samples. These results suggest that these can act as the potential virulent factors in inducing food poisoning.

The pervasiveness of prophages in most bacterial genomes has been reported through the genome mining and comparative genomics. Prophages were known to be one of the main sources of strain variation and genetic diversity related with the virulence of various bacterial pathogens, such as *S. aureus*, *E. coli*, *Salmonella enterica*, and *S. pyogenes*[28-32] and many phage associated with virulent strains encode powerful extracellular toxins, effector and regulatory proteins, adhesins, serum resistance factors and superantigens [33-39]. In addition, prophage induction and mobility could shift bacterial diversities, and increase the dissemination of antibiotic resistance genes and other mobile genetic elements including pathogenicity islands in *S. aureus*, resulting in promoting bacterial evolution [40-44]. It is reported that liquid manure of swine can act as an important medium in transferring antibiotic resistance genes [45].

This study confirmed that gene families associated with “putative primase, superantigen-encoding pathogenicity islands SaPI”, “Integrase, superantigen-encoding pathogenicity islands SaPI” and “Broad-substrate range phospholipase C” were significantly enriched in pig. Normally, PIs are widely assumed to be mobile. However, thus far this mobility has been demonstrated directly only for *S. aureus* and *V. cholerae*. In both cases the transfer was phage-mediated [46-48], indicating that there is high possibility to transfer the SaPIs between microorganisms in pig than chicken samples.

In this study, the gene families related with “Biofilm formation” were highly enriched in pig samples than chicken samples at the level 3 SEED subsystem. The gene families associated with “Biofilm_Adhesin_biosynthesis”, “Biofilm formation in *Staphylococcus*”, “Protein YjgK cluster linked to biofilm formation” and “Symbiotic colonization and sigma-dependent biofilm formation gene cluster” were highly enriched in pig samples, although the gene families related with “Biofilm_Adhesin_biosynthesis” tended to present in both pig and chicken samples. Most of the microorganisms are present in nature by attaching to lifeless surfaces and growing. They are commonly used to grow on free-living (planktonic) state as free-floating in liquid. If suspended cells attach many kinds of surfaces including soil and aquatic environment, they will form biofilm and survive. The ability of a pathogen to form biofilm can protect them from host defenses and environmental stress [49]. The Formation of biofilm is stimulated from quorum sensing which has communication systems. Furthermore, quorum sensing can induce not only biofilm formation but also the secretion of virulence factors [50]. It has been reported that 80% of microbial infections are related to biofilm mediated by quorum sensing such as *E. coli*, *Salmonella*, *S. aureus*, *Campylobacter*, *Listeria monocytogenes* and *B. cereus* [51, 52]. Quorum sensing was reported to be involved with both bacterial food spoilage process and the pathogenicity of bacteria. Therefore, it has possibility to increase the risk of food poisoning. In addition, the quorum sensing plays the important roles in enhancing bacterial resistant to environment, antibiotics and the host immune system [53]. Especially, quorum sensing found in this study, was mediated by autoinducer-2 (AI-2). AI-2 mediated quorum sensing has also been confirmed to be involved in the *E. coli* and *Salmonella* typhimurium biofilm formation [54-57]. Furthermore, several studies have reported that quorum sensing also plays an important regulatory role in the formation and resuscitation of bacterial viable but non- culturable (VBNC) states [58], indicating that there are possibility that *E. coli* and *S*. typhimurium could constitute the biofilm in pig and chicken samples.

Antibiotic resistant bacteria has become an important public issue worldwide [59]. Antibiotic resistance genes could be detected in various environments such as animal feces and soil [60-63]. As such, the gene families related with the resistance to antibiotics, such as Fluoroquinolones, Methicillin, Beta-lactamase, Aminoglycoside, Vancomycin, Erythromycin and Streptothricin were identified in both pig and chicken samples in this study. It has been reported that Antibiotic resistance genes could significantly increase because of mobile genetic elements through conjugation, transduction, and transformation [64]. This study also detected the gene families associated with the transposable elements in pig and chicken samples. Therefore, the results of this study speculated that the presence of both antimicrobial resistance genes and mobile genetic elements in pigs and chicken samples could pose threat to the public health by spreading antimicrobial resistance among the human, livestock, and environments.

In this study, the gene families related with “Fibronectin/fibrinogen-binding protein” were highly enriched in pig than chicken samples. Fibronectin/fibrinogen-binding proteins are the subgroups of protein “Microbial Surface Components Recognizing Adhesive Matrix Molecule(s) (MSCRAMMs)”. Microbial Surface Components Recognizing Adhesive Matrix Molecule(s) (MSCRAMMs) are synthesized by many pathogenic Gram-positive and Gram-negative bacteria. It has been well known to contribute to the bacterial infection. Fibronectin binding proteins (FNBPs) are members of the MSCRAMM family [65]. *C. jejuni* CadF and FlpA FNBPs encoding fibronectin-binding proteins, two of adhesin proteins facilitate bacterial colonization and contribute to illness in a disease. *S. aureus* also has fibronectin-binding proteins (FnbA and FnbB) [66, 67], and possess serine-aspartate repeat family proteins (SdrC, SdrD and SdrE) [68-70] and clumping factors (ClfA and ClfB) [71, 72], which mediate staphylococcal adherence to components of the extracellular matrix of the host, suggesting that adhesion in *campylobacter* and *staphylococcus* contribute to biofilm formation or invasion. These proteins protect the pathogens from the extreme environment stress, and also are associated with pathogenesis, antibiotic and multidrug resistance. This study suggests that those factors can act as the potential risk factors for pathogen colonization in food and outbreak of food poisoning.

## Supplemental Materials

Supplementary data for this paper are available on-line only at http://jmb.or.kr.

## References

[1] Oliver SP, Jayarao BM, Almeida RA (2005). Foodborne pathogens in milk and the dairy farm environment: food safety and public health implications. Foodbourne Pathog. Dis..

[2] Kreling V, Falcone FH, Kehrenberg C, Hensel A (2020). *Campylobacter* sp.: pathogenicity factors and prevention methods-new molecular targets for innovative antivirulence drugs?. Appl. Microbiol. Biotechnol..

[3] Weidenmaier C, Kokai-Kun JF, Kristian SA, Chanturiya T, Kalbacher H, Gross M (2004). Role of teichoic acids in *Staphylococcus aureus* nasal colonization, a major risk factor in nosocomial infections. Nat. Med..

[4] Wertheim HFL, Walsh E, Choudhurry R, Melles DC, Boelens HAM, Miajlovic H (2008). Key role for clumping factor B in *Staphylococcus aureus* nasal colonization of humans. PLoS Med..

[5] McDermott P, Zhao S, Wagner D, Simjee S, Walker R, White D (2002). The food safety perspective of antibiotic resistance. Anim. Bbiotechnol..

[6] Gomez-Alvarez V, Teal TK, Schmidt TM (2009). Systematic artifacts in metagenomes from complex microbial communities. ISME J..

[7] Cox MP, Peterson DA, Biggs PJ (2010). SolexaQA: At-a-glance quality assessment of Illumina second-generation sequencing data. BMC Bioinformatics.

[8] Douterelo I, Calero-Preciado C, Soria-Carrasco V, Boxall JB (2018). Whole metagenome sequencing of chlorinated drinking water distribution systems. Environ. Sci. Water Res. Technol..

[9] Hacker J, Kaper JB (2000). Pathogenicity islands and the evolution of microbes. AIMS Microbiol..

[10] Bintsis T (2017). Foodborne pathogens. AIMS Mcrobiol..

[11] Hazards EPoB (2010). Scientific opinion on quantification of the risk posed by broiler meat to human campylobacteriosis in the EU. EFSA J..

[12] Doyle MP, Erickson MC (2006). Reducing the carriage of foodborne pathogens in livestock and poultry. Poultry Sci..

[13] Heredia N, García S (2018). Animals as sources of food-borne pathogens: a review. Anim. Nutr..

[14] Gerlach RG, Hensel MJIJoMM (2007). Protein secretion systems and adhesins: the molecular armory of Gram-negative pathogens. Int. J. Med. Microbiol..

[15] Green ER, Mecsas J (2016). Bacterial secretion systems: an overview. Microbiol. Spectr..

[16] Kanonenberg K, Schwarz CK, Schmitt LJRim (2013). Type I secretion systems-a story of appendices. Res. Microbiol..

[17] Piddock LJJNRM (2006). Multidrug-resistance efflux pumps? not just for resistance. Nat. Rev. Microbiol..

[18] Cianciotto NPJTim (2005). Type II secretion: a protein secretion system for all seasons. Trends Microbiol..

[19] Filloux AJBeBA-MCR (2004). The underlying mechanisms of type II protein secretion. Biochimica et Biophysica Acta (BBA)-Mol. Cell Res..

[20] Cascales EJEr (2008). The type VI secretion toolkit. EMBO Rep..

[21] Filloux A, Hachani A, Bleves SJM (2008). The bacterial type VI secretion machine: yet another player for protein transport across membranes. Microbiology.

[22] Shrivastava S, Mande SSJPo (2008). Identification and functional characterization of gene components of Type VI secretion system in bacterial genomes. PLoS One.

[23] Korotkov KV, Sandkvist M, Hol WG (2012). The type II secretion system: biogenesis, molecular architecture and mechanism. Nat. Rev. Microbiol..

[24] Galán JE, Wolf-Watz HJN (2006). Protein delivery into eukaryotic cells by type III secretion machines. Nature.

[25] Cornelis GRJNRM (2006). The type III secretion injectisome. Nat. Rev. Microbiol..

[26] Büttner DJM, Reviews MB (2012). Protein export according to schedule: architecture, assembly, and regulation of type III secretion systems from plant-and animal-pathogenic bacteria. Microbiol. Mol. Biol. Rev..

[27] Pieper R, Zhang Q, Parmar PP, Huang ST, Clark DJ, Alami H (2009). The *Shigella dysenteriae* serotype 1 proteome, profiled in the host intestinal environment, reveals major metabolic modifications and increased expression of invasive proteins. Proteomics.

[28] Cleary P, LaPenta D, Vessela R, Lam H, Cue D (1998). A globally disseminated M1 subclone of group A streptococci differs from other subclones by 70 kilobases of prophage DNA and capacity for high-frequency intracellular invasion. INFIBR.

[29] Aziz RK, Edwards RA, Taylor WW, Low DE, McGeer A, Kotb M (2005). Mosaic prophages with horizontally acquired genes account for the emergence and diversification of the globally disseminated M1T1 clone of *Streptococcus pyogenes*. J. Bacteriol. Res..

[30] Goerke C, Pantucek R, Holtfreter S, Schulte B, Zink M, Grumann D (2009). Diversity of prophages in dominant *Staphylococcus aureus* clonal lineages. J. Bacteriol. Res..

[31] Mead PS, Griffin PM (1998). *Escherichia coli* O157: H7. Lancet.

[32] Figueroa‐Bossi N, Uzzau S, Maloriol D, Bossi L (2001). Variable assortment of prophages provides a transferable repertoire of pathogenic determinants in *Salmonella*. Mol. Microbiol..

[33] Cooke FJ, Wain J, Fookes M, Ivens A, Thomson N, Brown DJ (2007). Prophage sequences defining hot spots of genome variation in *Salmonella enterica* serovar Typhimurium can be used to discriminate between field isolates. Clin. Microbiol. Newsl..

[34] Rahimi F, Bouzari M, Katouli M, Pourshafie MR (2012). Prophage and antibiotic resistance profiles of methicillin-resistant *Staphylococcus aureus* strains in Iran. Arch. Virol..

[35] Thomson N, Baker S, Pickard D, Fookes M, Anjum M, Hamlin N (2004). The role of prophage-like elements in the diversity of *Salmonella enterica* serovars. J. Mol. Biol..

[36] Bae T, Baba T, Hiramatsu K, Schneewind OJMm (2006). Prophages of *Staphylococcus aureus* Newman and their contribution to virulence. Mol. Microbiol..

[37] Hermans AP, Abee T, Zwietering MH, Aarts HJ (2005). Identification of novel *Salmonella enterica* serovar Typhimurium DT104-specific prophage and nonprophage chromosomal sequences among serovar Typhimurium isolates by genomic subtractive hybridization. Appl. Environ..

[38] Banks DJ, Beres SB, Musser JMJTim (2002). The fundamental contribution of phages to GAS evolution, genome diversification and strain emergence. Trends Microbiol..

[39] Ohnishi M, Kurokawa K, Hayashi TJTim (2001). Diversification of *Escherichia coli* genomes: are bacteriophages the major contributors?. Trends Microbiol..

[40] Chen J, Novick RP (2009). Phage-mediated intergeneric transfer of toxin genes. Science..

[41] Duerkop BA, Clements CV, Rollins D, Rodrigues JL, Hooper LVJPotNAoS (2012). A composite bacteriophage alters colonization by an intestinal commensal bacterium. Proc Natl Acad Sci U S A.

[42] Mills S, Shanahan F, Stanton C, Hill C, Coffey A, Ross RPJGm (2013). Movers and shakers: influence of bacteriophages in shaping the mammalian gut microbiota. Gut Microbes.

[43] Úbeda C, Maiques E, Knecht E, Lasa Í, Novick RP, Penadés JRJMm (2005). Antibiotic‐induced SOS response promotes horizontal dissemination of pathogenicity island‐encoded virulence factors in staphylococci. Mol. Microbiol..

[44] Zhang Y, LeJeune JTJVm (2008). Transduction of blaCMY-2, tet (A), and tet (B) from *Salmonella enterica* subspecies enterica serovar Heidelberg to S. Typhimurium. Vet. Microbiol..

[45] Yang F, Han B, Gu Y, Zhang KJSr (2020). Swine liquid manure: A hotspot of mobile genetic elements and antibiotic resistance genes. Sci. Rep..

[46] Karaolis DK, Johnson JA, Bailey CC, Boedeker EC, Kaper JB, Reeves PRJPotNAoS (1998). A *Vibrio cholerae* pathogenicity island associated with epidemic and pandemic strains. Proc. Natl. Acad. Sci. USA.

[47] Lindsay JA, Ruzin A, Ross HF, Kurepina N, Novick RPJMm (1998). The gene for toxic shock toxin is carried by a family of mobile pathogenicity islands in *Staphylococcus aureus*. Mol. Microbiol..

[48] O'Shea YA, Boyd EFJFml (2002). Mobilization of the Vibrio pathogenicity island between *Vibrio cholerae* isolates mediated by CP-T1 generalized transduction. FEMS Microbiol. Lett..

[49] Stewart PSJIjomm (2002). Mechanisms of antibiotic resistance in bacterial biofilms. Int. J. Med. Microbiol. Suppl..

[50] Dufour D, Leung V, Lévesque CMJET (2010). Bacterial biofilm: structure, function, and antimicrobial resistance. Endod Topics.

[51] Arciola CR, Campoccia D, Speziale P, Montanaro L, Costerton JWJB (2012). Biofilm formation in Staphylococcus implant infections. A review of molecular mechanisms and implications for biofilm-resistant materials. Biomaterials.

[52] Machado I, Silva LR, Giaouris ED, Melo LF, Simões MJFRI (2020). Quorum sensing in food spoilage and natural-based strategies for its inhibition. Int. Food Res. J..

[53] Galie S, García-Gutiérrez C, Miguélez EM, Villar CJ, Lombó FJFim (2018). Biofilms in the food industry: health aspects and control methods. Front. Microbiol..

[54] Pereira CS, Santos AJ, Bejerano‐Sagie M, Correia PB, Marques JC, Xavier KBJMm (2012). Phosphoenolpyruvate phosphotransferase system regulates detection and processing of the quorum sensing signal autoinducer‐2. Mol. Microbiol..

[55] Khan F, Javaid A, Kim Y-MJCdt (2019). Functional diversity of quorum sensing receptors in pathogenic bacteria: Interspecies, intraspecies and interkingdom level. Curr. Drug Targets.

[56] Xue T, Zhao L, Sun H, Zhou X, Sun BJCr (2009). LsrR-binding site recognition and regulatory characteristics in *Escherichia coli* AI-2 quorum sensing. Cell Res..

[57] Herzberg M, Kaye IK, Peti W, Wood TKJJob (2006). YdgG (TqsA) controls biofilm formation in *Escherichia coli* K-12 through autoinducer 2 transport. J. Bacteriol. Res..

[58] Li J, Zhao XJFRI (2020). Effects of quorum sensing on the biofilm formation and viable but non-culturable state. Int. Food Res. J..

[59] Chagas TPG, Seki L, Cury J, Oliveira J, Dávila A, Silva D (2011). Multiresistance, beta‐lactamase‐encoding genes and bacterial diversity in hospital wastewater in Rio de Janeiro, Brazil. J. Appl. Microbiol..

[60] Zhu Y-G, Johnson TA, Su J-Q, Qiao M, Guo G-X, Stedtfeld RD (2013). Diverse and abundant antibiotic resistance genes in Chinese swine farms. Proc. Natl. Acad. Sci. U S A.

[61] Li B, Yang Y, Ma L, Ju F, Guo F, Tiedje JM (2015). Metagenomic and network analysis reveal wide distribution and co-occurrence of environmental antibiotic resistance genes. ISME J..

[62] Wu N, Qiao M, Zhang B, Cheng WD, Zhu YG (2010). Abundance and diversity of tetracycline resistance genes in soils adjacent to representative swine feedlots in China. Environ. Sci. Technol..

[63] Marti R, Scott A, Tien Y-C, Murray R, Sabourin L, Zhang Y (2013). Impact of manure fertilization on the abundance of antibioticresistant bacteria and frequency of detection of antibiotic resistance genes in soil and on vegetables at harvest. Appl. Environ..

[64] Zhang X-X, Zhang TJEs, technology (2011). Occurrence, abundance, and diversity of tetracycline resistance genes in 15 sewage treatment plants across China and other global locations. Environ. Sci. Technol..

[65] Kang M, Ko Y-P, Liang X, Ross CL, Liu Q, Murray BE (2013). Collagen-binding microbial surface components recognizing adhesive matrix molecule (MSCRAMM) of Gram-positive bacteria inhibit complement activation via the classical pathway. J Biol Chem.

[66] McCourt J, O'Halloran DP, McCarthy H, O'Gara JP, Geoghegan JAJFml (2014). Fibronectin-binding proteins are required for biofilm formation by community-associated methicillin-resistant *Staphylococcus aureus* strain LAC. FEMS Microbiol. Lett..

[67] O'Neill E, Pozzi C, Houston P, Humphreys H, Robinson DA, Loughman A (2008). A novel *Staphylococcus aureus* biofilm phenotype mediated by the fibronectin-binding proteins, FnBPA and FnBPB. J. Bacteriol. Res..

[68] Josefsson E, McCrea KW, Eidhin DN, O'Connell D, Cox J, Hook M (1998). Three new members of the serine-aspartate repeat protein multigene family of *Staphylococcus aureus*. Microbiology.

[69] Corrigan RM, Foster TJJP (2009). An improved tetracycline-inducible expression vector for Staphylococcus aureus. Plasmid.

[70] O' Brien L, Kerrigan SW, Kaw G, Hogan M, Penadés J, Litt D (2002). Multiple mechanisms for the activation of human platelet aggregation by *Staphylococcus aureus*: roles for the clumping factors ClfA and ClfB, the serine-aspartate repeat protein SdrE and protein A. Mol. Microbiol..

[71] Ní Eidhin D, Perkins S, Francois P, Vaudaux P, Höök M, Foster TJJMm (1998). Clumping factor B (ClfB), a new surface‐located fibrinogen‐binding adhesin of *Staphylococcus aureus*. Mol. Microbiol..

[72] McDevitt D, Francois P, Vaudaux P, Foster TJMm (1994). Molecular characterization of the clumping factor (fibrinogen receptor) of *Staphylococcus aureus*. Mol. Microbiol..

