# Supplementary material for: Exploring the Microbial Community and Functional Characteristics of the Livestock Feces Using the Whole Metagenome Shotgun Sequencing
Source: J Microbiol Biotechnol. 2022 Dec 16;33(1):51–60. doi: 10.4014/jmb.2209.09013 (PMC9896000; doi:10.4014/jmb.2209.09013)
Supplement: Supplementary file 1 [file jmb-33-1-51-supple.pdf]

J. Microbiol. Biotechnol.
https://doi.org/10.4014/jmb.2209.09013

Supplementary Table

Supplementary Table 1. The livestock feces sample information

Species	Sample	location		Sampling date	Country & Province
		latitude	longitude		
Chicken 1	Feces	36.680083	127.196251	2019.09	South Korea Chungcheong
Chicken 2	Feces				
Chicken 3	Feces				
Pig 1	Feces	36.523742	126.708225	2020.06	South Korea Chungcheong
Pig 2	Feces				
Pig 3	Feces				

Supplementary Table S2. Raw data statistics of sequences produced by Illumina NovaSeq platform

Sample ID	Total read bases (bp)	Total reads	GC (%)	AT (%)	Q20 (%)	Q30 (%)
Chicken1	8,041,014,250	53,251,750	48.63	51.37	96.11	90.05
Chicken2	8,798,979,890	58,271,390	48.43	51.57	96.14	90.02
Chicken3	8,043,272,002	53,266,702	40.88	51.12	96.49	90.88
Pig1	8,095,585,952	53,613,152	40.88	59.12	96.13	89.89
Pig2	8,038,421,882	53,234,582	40.88	59.92	96.12	89.84
Pig3	8,448,876,726	55,952,826	45.13	54.87	96.3	90.42

GC(%) : GC content; AT(%) : AT content; Q20(%) : Ratio of bases that have phred quality score of over 20; Q30(%) : Ratio of bases that have phred quality score of over 30

Supplementary Table S3. Number of sequence reads, length of contigs, number of contig, identified protein features based on shotgun metagenomics data analyzed using MG-RAST

SAMPLE ID	MG-RAST ID	Length of contig(bp)		Number of contigs		Alignment: Identified Protein Features
		Pre-QC	Post-QC	Pre-QC	Post-QC	
Chicken1	mgs849758	100,627,623	100,468,022	385,802	385,239	188,368
Chicken2	mgs849761	118,189,068	117,989,051	456,636	455,927	202,208
Chicken3	mgs849764	155,540,997	155,305,573	598,918	598,067	289,922
Pig1	mgs849743	149,993,850	143,352,593	592,979	566,654	210,503
Pig2	mgs849746	155,757,312	149,371,840	612,008	586,884	229,230
Pig3	mgs849749	182,844,069	175,490,667	723,695	694,641	293,637

QC: quality control
